# Lane-Level Map-Matching Method for Vehicle Localization Using GPS and Camera on a High-Definition Map

**DOI:** 10.3390/s20082166

**Published:** 2020-04-11

**Authors:** Jeong Min Kang, Tae Sung Yoon, Euntai Kim, Jin Bae Park

**Affiliations:** 1 Department of Electrical and Electronic Engineering, Yonsei University, Seoul 03722, Korea; kangjm@yonsei.ac.kr (J.M.K.); etkim@yonsei.ac.kr (E.K.); 2 Department of Electrical Engineering, Changwon National University, Changwon 51140, Korea; tsyoon@changwon.ac.kr

**Keywords:** autonomous driving, localization, lane detection, GNSS, HD map, map-matching

## Abstract

Accurate vehicle localization is important for autonomous driving and advanced driver assistance systems. Existing precise localization systems based on the global navigation satellite system cannot always provide lane-level accuracy even in open-sky environments. Map-based localization using high-definition (HD) maps is an interesting method for achieving greater accuracy. We propose a map-based localization method using a single camera. Our method relies on road link information in the HD map to achieve lane-level accuracy. Initially, we process the image—acquired using the camera of a mobile device—via inverse perspective mapping, which shows the entire road at a glance in the driving image. Subsequently, we use the Hough transform to detect the vehicle lines and acquire driving link information regarding the lane on which the vehicle is moving. The vehicle position is estimated by matching the global positioning system (GPS) and reference HD map. We employ iterative closest point-based map-matching to determine and eliminate the disparity between the GPS trajectories and reference map. Finally, we perform experiments by considering the data of a sophisticated GPS/inertial navigation system as the ground truth and demonstrate that the proposed method provides lane-level position accuracy for vehicle localization.

## 1. Introduction

As increasing research on autonomous driving and advanced driver assistance systems (ADAS) is being conducted, more precise vehicle localization is required. Among the many challenges involved regarding the development of a reliable ADAS, the localization for determining the current position of a vehicle is a fundamental requirement for these systems. In fact, systems wherein; the driving lane is crucial, such as the lane keeping assist system, require lane-level accuracy.

To obtain accurate position information in an outdoor environment, an autonomous vehicle is equipped with a set of sensors to collect ambient information. The most widely used affordable sensor for providing a contemporary position of the vehicle is a global navigation satellite system (GNSS), such as the global positioning system (GPS). The primary advantages of this system are that it provides the global position of the vehicle, and errors are not accumulated. However, a single-point position accuracy of the GNSS does not improve beyond 2 to 3 m in an open-sky environment with no degraded signals [1]. Easy techniques to overcome these drawbacks involve exploiting the additional infrastructures, such as the differential global positioning system (DGPS), carrier-phase DGPS, and real-time kinematic GPS. These techniques utilize a base station system to improve the accuracy of the global position of a mobile station by reducing errors using a certain type of correction signal. However, in the case of autonomous driving based only on GNSS, the performance of position accuracy cannot provide high-level position accuracy in a signal-degraded environment owing to signal outages, multipath issues, and poor sky view. Even the most sophisticated system cannot guarantee lane-level accuracy and continuous positioning; consequently, these challenges present problems regarding reliability in a precise ADAS.

To overcome the drawback of the GNSS operation, a method using an additional sensor is considered. The current localization approaches for autonomous driving can be classified according to the type of the sensor, i.e., active or passive. Active sensor approaches, which utilize a light detection and ranging (LiDAR) sensor, are extensively employed to recognize information from the environment, such as curbs, road shapes, rails, vehicles, and road infrastructures. LiDAR components emit and receive laser signals to directly measure the distance and intensity from the sensor to the objects. This sensor can provide a 3D representation of the surrounding environment and does not depend on the lighting conditions. Another advantage is that this system can perform detection up to a distance of several hundred meters via installation of the sensor on top of the vehicle to cover 360° of the environment [2,3,4]. Based on these characteristics, several studies were conducted to utilize the LiDAR for autonomous driving systems. First, A 3D point cloud matching method that incorporates the use of local dynamic map was proposed [5]. This method used the probabilistic infrared intensity collected over multiple courses of the environment, and the probabilistic map increased the robustness to changes in the environment. Second, s method was proposed to detect curbs and road markings to create a feature map of the environment following which localization was performed within the created map [6]. This method identified the position of the vehicle using two features within the map and reduced the lateral and longitudinal errors to 0.3 m in an urban environment. Another approach was conducted on LiDAR-based road marking detection. Lane markings were extracted using the difference in the intensity between the asphalt and the ink painting from the ground data obtained using LiDAR [7,8]. This information was utilized to build a lane marking map and applied to the localization process. Another method was proposed that applied an alternative grid representation of the ground. This method improved the performance without increasing the computational requirements and reflectivity calibrations [9]. In another approaches, 3D LiDAR-based simultaneous localization and mapping (SLAM) methods such as lidar odometry and mapping (LOAM) and lightweight and ground-optimized lidar odometry and mapping (LeGO-LOAM) were proposed [10,11]. These methods provided a robust real-time six degree-of-freedom LiDAR odometry and mapping. In addition to LiDAR, other sensing methods such as inertial measurement unit (IMU) and wheel odometry are also equipped for vehicle localization in an urban environment. In another study, a vehicle position was estimated via particle filter and data fusion, and the lateral and longitudinal errors were reduced to a few centimeters on a 1.9 km long route [12]. While LiDAR can offer robust and accurate localization, it also presents certain shortcomings. That requires high power and is limited by high implementation costs as well as performance sensitivity to environmental conditions. While considering the case of high power, the Velodyne 64 system requires as much as 60 W of power. The large capacity of a raw point cloud data, which occupies hundreds of megabytes per kilometer, also places a burden on data processing.

Passive sensor approaches, such as vision-based techniques with cameras, are extensively used as a low-cost alternative for LiDAR. The device is easy to mount on a vehicle, changes in its hardware structure are not required, and it is considerably inexpensive when compared to LiDAR. Moreover, it can conjugate color and texture of all objects in the frame. As methods using the camera to alternative the GPS localization, numerous vision-based SLAM methods were proposed [13,14,15]. These methods calculated the odometry from the feature points of the image, and positioning in real-time. However, these vision-based SLAM methods could not provide robust performance in a complicated urban environment. In the complex environment, most autonomous vehicles use the front cameras to acquire frontal environment information. More recently, numerous studies on lane-level accuracy were conducted to apply the ADAS system [16,17]; in particular, many methods to estimate the location of a vehicle by detecting road or lane markings were developed [18,19,20,21,22,23,24]. A pair of cameras can recover the distance to a detected feature point. This method uses stereo vision provided by two cameras and produces the same kind of information as a LiDAR. The computational cost required to process the data using the stereo vision algorithm is lesser than that based on LiDAR. A visual odometry method, which uses camera information to calculate the trajectory of the vehicle, is one of the approaches utilizing a passive sensor [25]. The pose of a vehicle is calculated from the tracked feature points between the right and the left cameras mounted at the front of a vehicle; moreover, a random sample consensus algorithm, which can reject outlier estimates, is used. Certain research used stereo vision to calculate the 3D position of road lane markings which was computed using a stereo vision algorithm, and 3D features were used to create a 3D map that was applied for localization in real time [26,27]. However, the camera is a passive sensor that requires illumination; in particular, stereo vision has a smaller field of view (FOV) and less dense information when compared to LiDAR. Moreover, the stereo vision algorithm requires more computational power to improve accuracy. These shortcomings result in difficulty in guaranteeing steady performance; therefore, studies to analyze the improvements while integrating GPS and IMU were conducted [28,29]. In these studies, the camera recognized the lane markings and the GPS and IMU provided global positioning. However, further research is still required for changes in the image processing techniques and determining the magnitude of errors in various environments.

Another approach for vehicle localization is to use a map-matching method using a precise high-definition (HD) map. Map-based localization using a HD map is an interesting method to increase the robustness and reliability of the autonomous driving algorithm. A simple map-matching approach tailors the current position of the vehicle based on the nearest landmarks onto a vector representation of a road network, where point-to-point and point-to-curve matching methods were proposed [30,31]. These methods were easy to implement; however, the amount of computation and location errors increase as the map becomes more complex. To overcome this drawback, a method for comparing a trajectory of the vehicle using a HD map was proposed [32]. The mutual influences of the GPS location in a trajectory, distance, and topology were calculated. This method achieved better accuracy, but at a higher computational cost. Advanced map-matching methods that applied a fuzzy theory [33] and a probabilistic map [34] were proposed. However, these methods still present drawbacks when the sampling rate is low. To determine the matching accuracy, a method was proposed to calculate the horizontal distance from the center of the road [35]. However, this method could not present the position error of each vehicle point. Therefore, a method suggesting the accuracy of each point that is based on an iterative closest point (ICP) algorithm utilizing only GPS trajectory and map information was proposed [36]. This method enabled calculation of the position errors via consideration of the information acquired by the high-end equipment as a ground truth. A low-cost sensor was used without additional sensors; however, the average error was 1.7 m, which could not provide the lane-level accuracy. Map-matching methods based on additional sensors were also presented. The localization task indicates the processing of sensor data using the information available in the map. In these approaches, feature maps and dense maps were utilized [37,38]. The feature maps decreased the computational time and enabled greater application of the localization to a real environment. The localization method was also proposed to detect features using landmarks [39]. Lane markings, which are one of the kinds of landmarks considered, are easy to detect using LiDAR or a camera. Therefore, research was conducted to detect the lane markings and apply them to a localization algorithm [40,41]. An image localization method based on virtual generalizing random access memory was proposed [40], which built a neural map from landmarks detected by a vision system. However, the average lateral error was 1.12 m, which did not satisfy the lane-level accuracy requirements for autonomous driving.

In this paper, we propose a lane-level map-matching method for vehicle localization using the GPS and camera on the HD map. Our method relies on road link information, which indicates the center of each lane in the HD map. The position of the vehicle is estimated by matching the GPS and the reference HD map; subsequently, the ICP-based map-matching method is used to eliminate the residual disparity. The experiments using the data of a state-of-the-art GPS/inertial navigation system (INS) as the ground truth demonstrated that the position accuracy of our method is sufficient for autonomous driving systems.

The proposed method herein can enhance the position accuracy, and the contributions are as follows:

- The primary contribution of this paper is the achievement of lane-level position accuracy using data from GPS, a camera, and a map, which can be applied in autonomous driving applications. In particular, the position of the vehicle is finally on the HD map, the proposed method does not diverge in a complicated environment.
- Another contribution is the enhancement of localization performance via usage of low-cost sensors. We used GPS and camera sensors of a smartphone and the information of a HD map that was provided in advance. It was considerably inexpensive when compared to the LiDAR approaches.

The remainder of this paper is organized as follows. The description of the overall system and the reference map is introduced in Section 2. The extraction of driving link information is detailed in Section 3, and the map-based localization method is described in Section 4. The experimental setup and results are shown in Section 5, and Section 6 discusses the results of the proposed method and concludes the paper.

## 2. Preliminaries

### 2.1. System Overview

**Figure 1:**
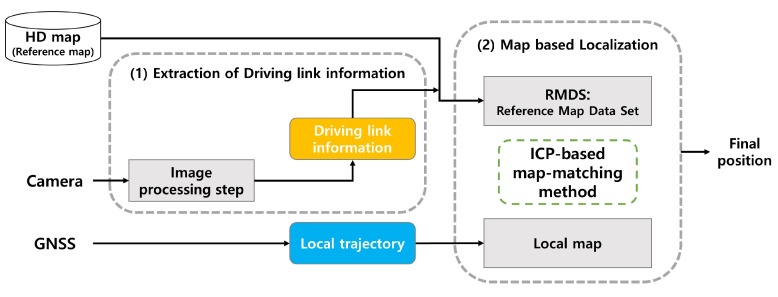
Framework of the proposed method.

The overall framework of the proposed method is depicted in Figure 1. Our approach involves two primary processes: (1) extraction of driving link information via image processing and (2) map-based localization. A vehicle travels in a predetermined direction on a lane on the right or left side of the entire road. In this paper, we define road link information, which indicates the center of each lane. A reference map consisting of the entire link map from the HD map is built using this information. In particular, we define the driving link information regarding the link on which the vehicle is currently driving in the entire road. The driving link information is extracted from the camera located at the front of the vehicle. The original image captured from the camera is swapped into a top-view image. In the image processing step, after the detection of all the vehicle lines from the top-view image, the driving link information is extracted from the detected information on vehicle lines. We set the information to 1 Hz in the same manner as the frequency of receiving GPS location information, since the position of the vehicle does not change abruptly. This frequency can be adjusted according to the period in which the location information is to be updated. From the driving link information, we extracted the reference map dataset (RMDS) in the map-based localization step. The RMDS is defined as the link map where the vehicle is located in the reference map. We build a local map from the vehicle trajectory acquired from the GPS. Based on the sliding window technique, we propose an ICP-based rigid map-matching method using both the local map and the RMDS, and finally estimate the position of the vehicle through the vehicle position on the map.

### 2.2. Description of the Reference Map

**Figure 2:**
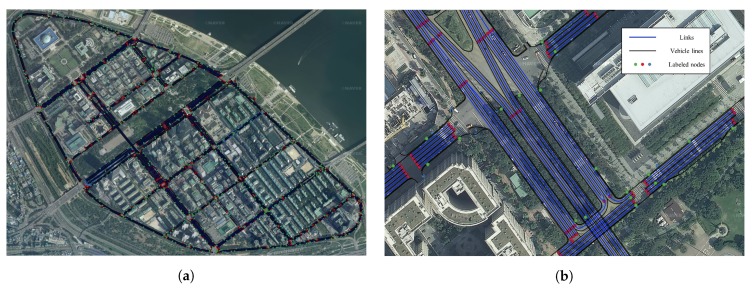
Example of precise roadmap: (**a**) precise roadmap in urban area; (**b**) magnified map.

As HD maps have become more precise, they have been applied and utilized in many studies related to autonomous driving. In addition to displaying roads and lanes accurately, structured layers contain more precise and various other information. A precise roadmap acquires high-precision data from a mobile mapping system (MMS) equipped with various sensors; moreover, it provides information regarding lanes, road facilities, signs, etc. with an error of only a few centimeters in various courses [42]. Therefore, this map can be used as a reference in the map-matching method. The geographical information system (GIS) is a useful tool for handling maps, and it involves presenting maps on a display to support various types of geographically related information. The maps can be represented using a shape file, which can be opened with professional GIS tools such as Quantum GIS (QGIS). The precise roadmap provided by the National Geographic Information Institute of South Korea using QGIS 3.4.11 is shown in Figure 2. Figure 2a shows the precise roadmap of an urban area; moreover, the map is formed from a road network composed of nodes and edges. Figure 2b shows the magnified map. In general, the vehicle does not deviate from the lane width while it travels on the road; the center of the vehicle is near the road link. Motivated by this, we defined the reference map as the link map. QGIS can be used to extract the position coordinates of the link as points and construct a reference map from these points. We further refined the reference map to achieve lane-level accuracy. Initially, as in a previous study, the driving azimuth is divided into east, west, south, and north [36]. Each link is stored separately from the median lane, which is a line indicated with safety signs, such as yellow solid lines and yellow dotted lines, or facilities [43]. In short, the reference map is prebuilt with azimuth and link information, and the RMDS is updated at each sampling time using the data from the GPS receiver. For simplicity, we denote the RMDS as $R_{k}^{m}$, where *k* denotes east, west, south or north, and m=1,2,3,… is the link information. For example, if the current driving lane is secondary in the west direction, $R_{west}^{2}$ is selected as the RMDS.

## 3. Extraction of Driving Link Information

To extract the driving link information, we utilized the vehicle lines in front of the vehicle. In our approach, the camera is used to search the link information of the vehicle. The camera is easy to integrate on a vehicle as it is smaller than other sensors and can acquire information from the front without requiring special calibration. The computer vision system for autonomous vehicles use cameras to detect lane markings in the range of up to 40 m. The driving link information is extracted by detecting all vehicle lines using image processing; then, it is transferred to the reference map for selecting RMDS. Details of the extraction of the link information is presented in the following sections.

### 3.1. Inverse Perspective Mapping

The first step in image processing is to generate the top view of the image. Images captured by the front-view camera have a perspective distortion in the direction of the optical axis. To remove this effect, we used inverse perspective mapping (IPM). IPM is a mathematical method that relates to coordinate systems with different perspectives. We eliminated the perspective effect in the images; consequently, the vehicle lines are parallel at the vertical direction. Further, we can specify a subregion of the image so that only the region of interest can be processed. IPM is the process used for obtaining the real-world coordinates of a point as image pixel coordinates.

Without loss of generality, any one pixel coordinate, which is denoted as p=(x,y), of an image projected in a 2D plane can be defined in a homogeneous coordinate, which is denoted by p=(ωx,ωy,ω). Here, ω denotes a non-zero constant. The mapping of the two points is then a projective transformation, and it can be expressed in the following forms:

(1) $$\omega \left(\begin{array}{c} x^{{}^{\prime }}\\ y^{{}^{\prime }}\\ 1 \end{array}\right)=\left(\begin{array}{ccc} h_{11} & h_{12} & h_{13}\\ h_{21} & h_{22} & h_{23}\\ h_{31} & h_{32} & 1 \end{array}\right)\left(\begin{array}{c} x\\ y\\ 1 \end{array}\right)\Rightarrow p^{{}^{\prime }}=Hp,$$

where, $p^{{}^{\prime }}$ and *p* represent the homogeneous coordinates of pixels in the original image and the projected image, respectively. This mapping is a linear transformation; moreover, the nonsingular matrix H is called the homogeneous transform matrix. The optimal estimate of the matrix H can be calculated by selecting matching pairs of pixels, $p^{{}^{\prime }}$ and *p*. The degree of freedom of the matrix in a 2D projection transformation is eight; finally, four matching pairs are required. The H matrix can be obtained by a direct linear transformation, and the equation is as follows [44]:

(2) $$\left(\begin{array}{cccccccc} x_{1} & y_{1} & 1 & 0 & 0 & 0 & -x_{1}x_{1}^{{}^{\prime }} & -y_{1}x_{1}^{{}^{\prime }}\\ 0 & 0 & 0 & x_{1} & y_{1} & 1 & -x_{1}y_{1}^{{}^{\prime }} & -y_{1}y_{1}^{{}^{\prime }}\\ x_{2} & y_{2} & 1 & 0 & 0 & 0 & -x_{2}x_{2}^{{}^{\prime }} & -y_{2}x_{2}^{{}^{\prime }}\\ 0 & 0 & 0 & x_{2} & y_{2} & 1 & -x_{2}y_{2}^{{}^{\prime }} & -y_{2}y_{2}^{{}^{\prime }}\\ x_{3} & y_{3} & 1 & 0 & 0 & 0 & -x_{3}x_{3}^{{}^{\prime }} & -y_{3}x_{3}^{{}^{\prime }}\\ 0 & 0 & 0 & x_{1} & y_{1} & 1 & -x_{3}y_{3}^{{}^{\prime }} & -y_{3}y_{3}^{{}^{\prime }}\\ x_{4} & y_{4} & 1 & 0 & 0 & 0 & -x_{4}x_{4}^{{}^{\prime }} & -y_{4}x_{4}^{{}^{\prime }}\\ 0 & 0 & 0 & x_{4} & y_{4} & 1 & -x_{4}y_{4}^{{}^{\prime }} & -y_{4}y_{4}^{{}^{\prime }} \end{array}\right)\left(\begin{array}{c} h_{11}\\ h_{12}\\ h_{13}\\ h_{21}\\ h_{22}\\ h_{23}\\ h_{31}\\ h_{32} \end{array}\right)=\left(\begin{array}{c} x_{1}^{{}^{\prime }}\\ y_{1}^{{}^{\prime }}\\ x_{2}^{{}^{\prime }}\\ y_{2}^{{}^{\prime }}\\ x_{3}^{{}^{\prime }}\\ y_{3}^{{}^{\prime }}\\ x_{4}^{{}^{\prime }}\\ y_{4}^{{}^{\prime }} \end{array}\right).$$

Once H is determined, all pixels of the image can be projected to a top-view image using the estimated H. The top-view image can be obtained by applying the homogeneous transform matrix to each pixel of the image. The equation is derived as follows:

(3) $$x_{i}^{{}^{\prime }}=\frac{h_{11}x_{i}+h_{12}y_{i}+h_{13}}{h_{31}x_{i}+h_{32}y_{i}+1},y_{i}^{{}^{\prime }}=\frac{h_{21}x_{i}+h_{22}y_{i}+h_{23}}{h_{31}x_{i}+h_{32}y_{i}+1}.$$

**Figure 3:**
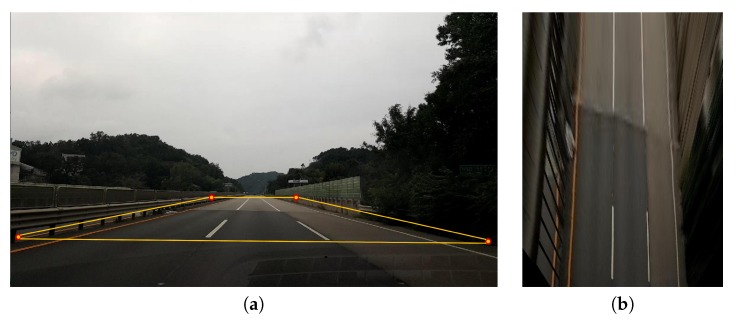
Sample inverse perspective mapping (IPM) image: (**a**) original image; (**b**) IPM image.

In the proposed method, we set the original image resolution of 1920 × 1080 pixels, and normalized the converted IPM image to a resolution of 720 × 1280 pixels. Figure 3 shows a sample IPM image, where the top-view image is obtained from the original image. Figure 3a shows the original image with the region of interest for extracting vehicle lines marked by a yellow box. Figure 3b shows the transformed IPM image. The vehicle lines appear as parallel straight lines in the IPM image.

### 3.2. Detection of Vehicle Lines

To extract the driving link information, we detected all vehicle lines from the top-view image. A candidate region was selected by designating the region of interest. The overall process for the detection of vehicle lines involves three steps, which are described in the following subsections.

#### 3.2.1. Adaptive Histogram Thresholding

**Figure 4:**
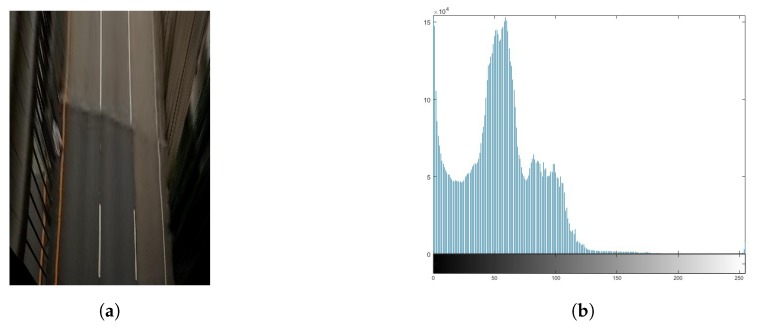
Histogram for region of interest: (**a**) region of interest image; (**b**) histogram.

The critical disadvantage of image processing is that it is dependent on the illumination conditions. If we select a fixed threshold value in the region of interest of the image, the binarization results for the gray level image can depend on the road conditions or changes in illumination. We proposed an adaptive histogram thresholding method based on the characteristic that the pixel value of the vehicle line appears on the histogram distribution of the entire image. After analyzing the histogram for the region of interest, we set the threshold value for the brightness corresponding to the pixels of the vehicle line. Figure 4 shows a example of the histogram for the region of interest. The histogram shows a high frequency for the road area and the second highest frequency for the vehicle lines. Therefore, we can convert a color image to a gray image that distinguishes the area of the vehicle lines from the other areas by setting a threshold value between the first peak and the second peak of the histogram.

#### 3.2.2. Edge Detection

After converting the color image to a gray image, we used an edge detection algorithm to extract the candidate vehicle lines. When the edge detection algorithm is directly applied to the gray image, the result of the vehicle line detection can be unstable owing to noise. Therefore, we performed a closing operation of the morphology to remove the noise. This operation also has the effect of removing small noise and retaining the candidate pixels of the vehicle lines. After performing the morphology operation, we used the Canny edge detector to detect the edges of the vehicle lines.

#### 3.2.3. Detection of Vehicle Lines Using Hough Transform

After Canny edge detection, we detected the vehicle lines. The vehicle lines are parallel in the vertical direction. This property is a constraint of the line detection while the Hough transform is used. Then, this method is used to identify straight lines in the edge image. The line is represented by the following equation:

(4) ρ=xcosφ+ysinφ,

where ρ is the distance between the line and the image origin, φ is the angle of the perpendicular to the line, and *x*, *y* are the points on the image coordinate. Therefore, Hough transform can generate a matrix that corresponds to ρ and φ.

**Figure 5:**
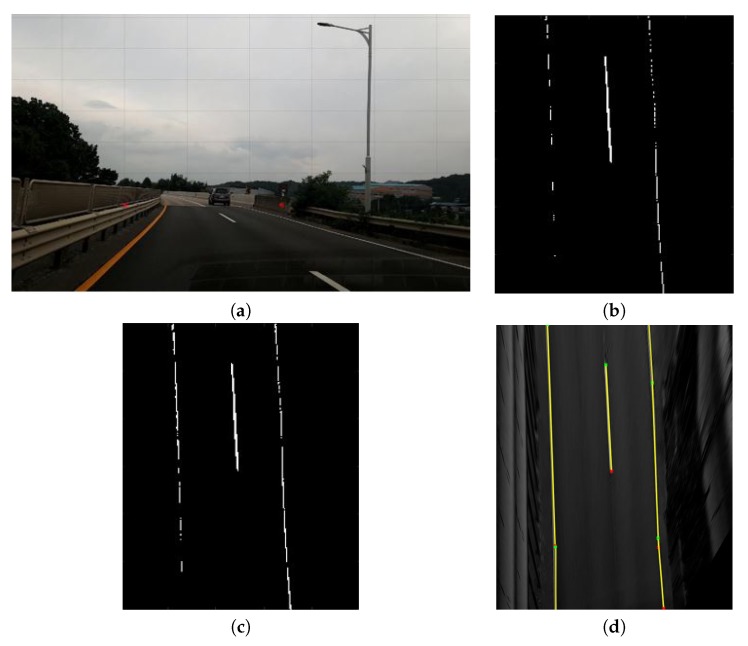
Examples of detection of vehicle lines: (**a**) original image; (**b**) image after edge detection; (**c**) image after edge operation; (**d**) image after vehicle line detection.

Figure 5 shows the examples for the detection of vehicle lines. From the top-view image of the region of interest in Figure 5a, the edges are extracted as shown in Figure 5b. Figure 5c shows the image obtained after edge operation. As it can be observed from the edge image, the process demonstrates a high response to the vehicle lines; thus, we can retain the highest values. Figure 5d shows the image after the detection of vehicle lines. In this example, we can detect all the vehicle lines from the top-view image.

### 3.3. Driving Link Information

In the proposed method, we defined driving link information as the link on which the vehicle is currently driving on the entire road. After all the vehicle lines from the top-view image were detected, the driving link information was extracted from the information of the detected vehicle lines.

#### 3.3.1. Link Information by Constraint

The driving link information is updated at a frequency of 1 Hz, which demonstrates the maximum frequency of image frame processing per second. As the situation changes while driving, the process demonstrates a limitation in obtaining steady information. Therefore, we propose a constrained extraction strategy for the driving link information. First, we set the candidate pixels that are separated by the interval of the lane width to detect the vehicle lines out of various lane markings. The typical lane width of the road is 3.0 to 3.25 m in Korea [45]. Second, we obtained the previous driving link information while assuming that no vehicle lines are detected on both sides in the image processing step, provided that the vehicle lines are always detectable on both sides of the vehicle and lane changes are not made drastically during driving. This can cope with line disconnection or false detection. Based on these constraints, the leftmost vehicle line indicates the first lane of the road. The current driving link information can be determined by calculating the number of vehicle lines from the leftmost line.

#### 3.3.2. Detection of Yellow Lane Marking

Highways and exclusive roads for vehicles have median facilities installed with barriers; however, roads in urban areas have yellow solid lines acting as a median lane. In this case, the vehicle lines on the opposite side of the road can also be detected. To solve this problem, we utilized the characteristic that the color of the median lane in urban roads is yellow; therefore, when a yellow lane is detected in the lines on the left of the direction in which the vehicle is driven, we recognize the line as the median lane and obtain the current driving link. The yellow lane in the image can be defined by a standard color, which is equivalent to the RGB value of (255, 183, 0). We can detect the yellow lane in YCbCr space, where Y represents the luminance component and Cb and Cr represent the chrominance components; moreover, the equation is as follows [46]:

(5) $$C_{cum}\left(k\right)=\sum_{\begin{array}{c} k=0 \end{array}}^{255}Hist\left(k\right),\;B\left(x,y\right)=\left\{\begin{array}{cc} 1, & if\;C_{cum}\left(I\left(x,y\right)\right)<T_{tr}\\ 0, & else, \end{array}\right.$$

Here, $C_{cum}$, Hist, *B*, *I*, and $T_{tr}$ are the cumulative histogram, histogram, binary image, intensity, and threshold, respectively.

**Figure 6:**
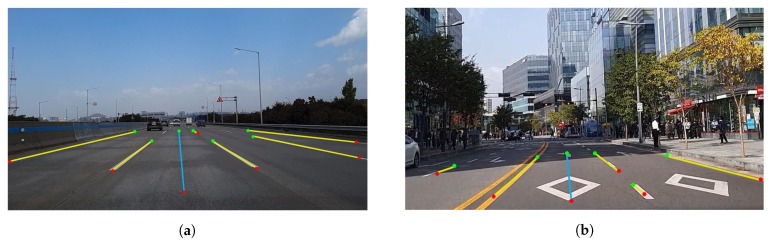
Example of extraction of driving link information: (**a**) highway area; (**b**) urban area.

Figure 6 shows examples of the driving link information from the image. The current driving link information is extracted from the detected vehicle lines as shown in Figure 6a. In the case of Figure 6b, where the vehicle lines in the opposite direction are detected, the current driving link information is extracted by the detection of yellow lane markings.

## 4. Map-Based Localization

In the map-based localization step, we built a local map using the GPS trajectory to describe the trajectory of a vehicle. The reference map is prebuilt with azimuth and link information, and the RMDS is updated at each sampling time instant from the image processing step. We used the RMDS to calculate a transformation matrix that contains a rotation matrix and translation vector between the local map and RMDS. We proposed an ICP-based rigid map-matching method to calculate the transformation matrix and estimate the final position. Details of the map-based localization are provided in the following sections.

### 4.1. Building a Local Map

**Figure 7:**
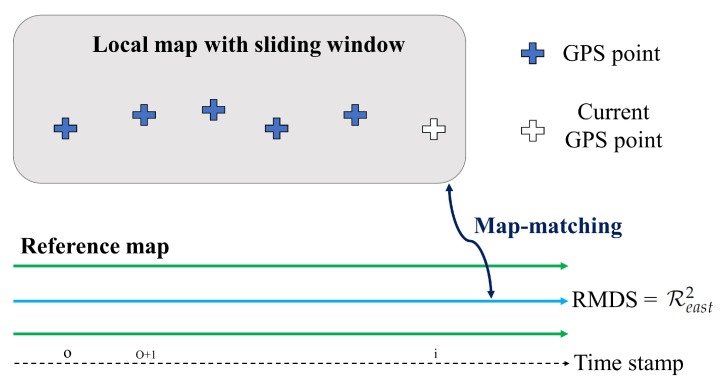
Composition of maps for map matching.

In general, a GPS device updates the data once per second. This information provides the global position of a vehicle; however, a current precise system based only on the GPS cannot always provide lane-level accuracy. To improve the position accuracy, a map-matching method is used. If the map-matching method uses an input map as the entire trajectory, only one transformation matrix is returned by the ICP algorithm; consequently, the disparity cannot be corrected. Therefore, we built a local map containing the position information from the GPS trajectory and performed ICP-based map-matching between the local map and the RMDS. The maps used for the matching method are shown in Figure 7. Using the built local map, we calculated the transformation matrix that is updated at each sampling time with the sliding window. The sliding window with the current position as the last timestamp helps to bound the part of the local map.

The GPS information used in the proposed method is position and azimuth, and the expression of the GPS point constituting the trajectory is as follows:

(6) $$v_{i}=\left(p_{xi},\;p_{yi},\;\phi_{i}\right),$$

where $p_{x}$ and $p_{y}$ represent the longitude and latitude coordinates, respectively, and $\phi_{i}$ is the azimuth. The current timestamp is *i*, and *o* is the first timestamp of the window. ϕ is the information used to extract the RMDS from the reference map; it is to be noted that only the position coordinates are utilized for map matching. Therefore, the state equation from *o* to *i* of the window timestamp can be defined as follows:

(7) $$x_{i}^{w}={\left[p_{xo},\;p_{yo},\;\;\ldots ,\;\;p_{xi},\;p_{yi}\right]}^{T}.$$

### 4.2. Iterative Closest Point-Based Rigid Map-Matching Method

As mentioned in Section 4.1, we built a local map and extracted the RMDS from ϕ. We can compute the transformation matrix between the local map and the RMDS based on the ICP algorithm, which is the most widely used and mature algorithm, which was created by Besl and McKay [47]. The key concept of the standard ICP algorithm is that it can compute correspondences between the two different point sets and calculate the transformation that minimizes the distance between the corresponding points. The algorithm can be forced to add a maximum matching threshold and can minimize the error function by iterative calculation. We denote the transformation matrix as $T_{L}^{R}$ between the local map and the RMDS; moreover, the error function *E* is defined as follows:

(8) $$E=\arg\min_{T_{L}^{R}}(\sum_{\begin{array}{c} i \end{array}}\|m_{i}-T_{L}^{R}x_{i}^{w}{\|}^{2}),$$

where $m_{i}$ represents the corresponding points of the RMDS. The transformation matrix $T_{L}^{R}$ consists of the rotation matrix $R_{L}^{R}$ and the translation vector $t_{L}^{R}$. The optimization can be solved after the iterative procedure. In the sliding window method, $R_{L}^{R}$ and $t_{L}^{R}$ are returned between each local map and RMDS, and the equation is as follows:

(9) $$R_{L}^{R}=\left(\begin{array}{cc} cos\theta_{e} & -sin\theta_{e}\\ sin\theta_{e} & cos\theta_{e} \end{array}\right),\;t_{L}^{R}=\left(\begin{array}{c} p_{xe}\\ p_{ye} \end{array}\right),$$

where $\theta_{e}$ is the rotational error, $p_{xe}$ and $p_{ye}$ are the translation errors of each axis.

In the proposed method, the local map shifts according to the sliding window. The ICP algorithm returns only one transformation between the local map and the RMDS; meanwhile, the algorithm is more sensitive to rotation than translation [48]. This results in the transformation not sufficiently reflecting the variance between the RMDS and the local map, which shifts over time. Therefore, we utilized a rigid body transformation to further reduce the transformation error. The new state equation ${\bar{x}}_{i}^{w}$ model that considers the scale is as follows:

(10) $${\bar{x}}_{i}^{w}={\bar{R}}_{L}^{R}x_{i}^{w}+{\bar{t}}_{L}^{R}=\left(\begin{array}{cc} s\cdot cos{\bar{\theta }}_{e} & -s\cdot sin{\bar{\theta }}_{e}\\ s\cdot sin{\bar{\theta }}_{e} & s\cdot cos{\bar{\theta }}_{e} \end{array}\right)x_{i}^{w}+\left(\begin{array}{c} {\bar{p}}_{xe}\\ {\bar{p}}_{ye} \end{array}\right),$$

where ${\bar{R}}_{L}^{R}$ and ${\bar{t}}_{L}^{R}$ are the new rotation matrix and the translation vector of the new transformation ${\bar{T}}_{L}^{R}$, respectively, and *s* is the scale factor.

Equation (10) can be expressed as data pairs, as presented below:

(11) $$\left(\begin{array}{cccc} p_{xo} & -p_{yo} & 1 & 0\\ p_{yo} & p_{xo} & 0 & 1\\ p_{xo+1} & -p_{yo+1} & 1 & 0\\ p_{yo+1} & p_{xo+1} & 0 & 1\\ \vdots & \vdots & \vdots & \vdots \\ p_{xi} & -p_{yi} & 1 & 0\\ p_{yi} & p_{xi} & 0 & 1 \end{array}\right)\left(\begin{array}{c} s\cdot cos{\bar{\theta }}_{e}\\ s\cdot sin{\bar{\theta }}_{e}\\ {\bar{p}}_{xe}\\ {\bar{p}}_{ye} \end{array}\right)=\left(\begin{array}{c} {\bar{p}}_{xo}\\ {\bar{p}}_{yo}\\ {\bar{p}}_{xo+1}\\ {\bar{p}}_{yo+1}\\ \vdots \\ {\bar{p}}_{xi}\\ {\bar{p}}_{yi} \end{array}\right).$$

Then, using the new system matrix, Equation (11) can be defined as follows:

(12) $$F_{i}\cdot {\bar{T}}_{L}^{R}={\bar{x}}_{i}^{w}.$$

Then, we can compute the estimation of the transformation ${\hat{T}}_{L}^{R}$ using the least squares method that minimizes the cost function *J*, where *J* is calculated as follows:

(13) $$J={\left({\bar{x}}_{i}^{w}-F_{i}{\hat{T}}_{L}^{R}\right)}^{T}\left({\bar{x}}_{i}^{w}-F_{i}{\hat{T}}_{L}^{R}\right).$$

We can compute the partial derivative to minimize the cost function *J* with respect to estimation of the transformation ${\hat{T}}_{L}^{R}$ as follows:

(14) $$\frac{\partial J}{\partial {\hat{T}}_{L}^{R}}=-{\left({\bar{x}}_{i}^{w}\right)}^{T}F_{i}-{\left({\bar{x}}_{i}^{w}\right)}^{T}F_{i}+2{\left({\hat{T}}_{L}^{R}\right)}^{T}F_{i}^{T}F_{i}=0.$$

Solving Equation (14), the final transformation estimation ${\hat{T}}_{i}^{R}$ is defined as follows:

(15) $${\hat{T}}_{L}^{R}={\left(F_{i}^{T}F_{i}\right)}^{-1}F_{i}^{T}{\bar{x}}_{i}^{w}.$$

From the final estimated rotation matrix ${\hat{R}}_{L}^{R}$ and the translation vector ${\hat{t}}_{L}^{R}$ of the final transformation, the final state can be derived as follows:

(16) $${\hat{x}}_{i}^{w}={\hat{R}}_{L}^{R}x_{i}^{w}+{\hat{t}}_{L}^{R}.$$

In this procedure, the last timestamp of ${\hat{x}}_{i}^{w}$ indicates the current position of the vehicle.

### 4.3. Vehicle Position on the Map

After the implementation of the ICP-based rigid map-matching method, the residual disparity is retained between the final state estimation and the RMDS. We used the vector projection theorem to eliminate this for the last timestamp of the estimation. The current vehicle position is located on the driving link of the HD map; then, the lane-level localization is performed.

## 5. Experimental Setup and Results

### 5.1. Introduction to the Experimental Setup

**Figure 8:**
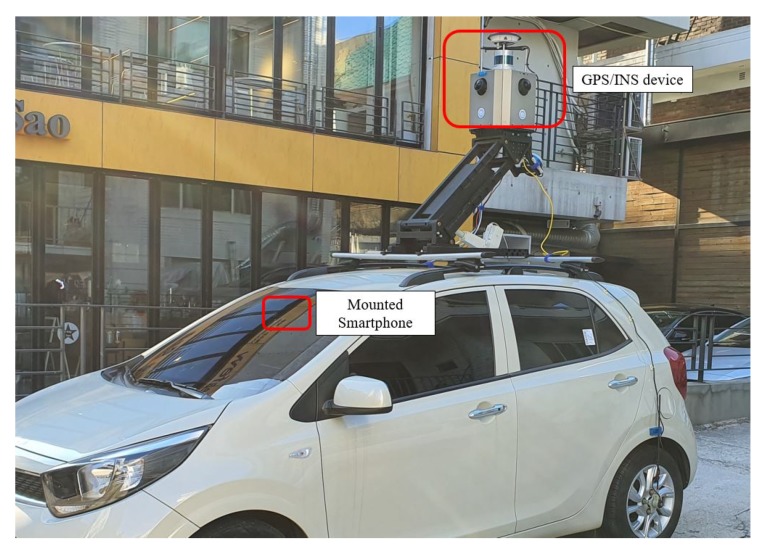
Setting of experimental devices.

The proposed method was tested in a real-world driving scenario. The primary purpose of our experiments was to verify that the position accuracy of the actual vehicle acquired using GPS and the captured images was effectively corrected by the proposed method. We utilized a smartphone mounted on the vehicle as an input sensor for the GPS and camera. A Samsung Galaxy Note 5 smartphone, which provided a horizontal accuracy of up to 10 m, was mounted in front of the rear-view mirror of the vehicle to obtain a desirable FOV. The processor of the mobile device was exynos 7420 octa-core (4 × 2.1 GHz Cortex-A57 and 4 × 1.5 GHz Cortex-A53). The sampling time of the GPS was set to 1 Hz, and the frames were captured at a frame rate of 15 fps at a resolution of 1920 × 1080 pixels, which were then sent for the image processing algorithm to process. We normalized the converted IPM image to a resolution of 720 × 1280 pixels. We also exploited a state-of-the-art navigation device consisting of GPS/INS to obtain the actual trajectory of the vehicle, which was considered as the ground truth. The sophisticated GPS/INS device was composed of the NovAtel/PwrPak7D-E1 [49] and NovAtel/VEXXIS GNSS-804 [50], which were rigidly installed in a box on the roof of the MMS vehicle. In this configuration, the device provided the position of the vehicle with an accuracy of 0.4 m while in motion and 0.02 m while at the stop. Thus, it was reasonable to assume that the vehicle position acquired by this device was the ground truth. The GPS/INS device and smartphone were positioned on the vehicle as shown in Figure 8. In this experiment, we used an MMS vehicle and only the sophisticated GPS/INS device of the vehicle to obtain the ground truth. We implemented and tested our method using an AMD Ryzen 7 3.59 GHz under Ubuntu 18.04 LTS laptop.

**Figure 9:**
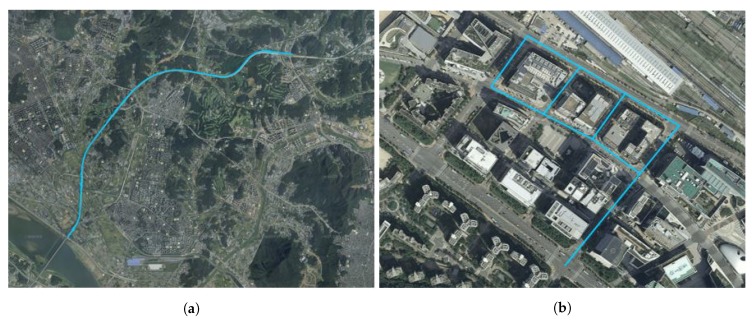
Overall map comprising of two courses: (**a**) course 1 (highway area); (**b**) course 2 (urban area).

We performed a test drive for the two courses shown in Figure 9. Figure 9a shows course 1, which has a 10.1 km highway area consisting of straight and smooth curves. Figure 9b shows course 2 in urban area, which has a total length of 2.3 km. The characteristics of the test courses are listed in Table 1. The number of GPS points, stored video length, and total number of frames for each course are listed.

### 5.2. Experimental Results

The evaluation of the proposed method was performed in three phases. The first phase involved an evaluation of the driving link information. We evaluated the extraction of the driving link information from the image processing stage for accurate RMDS update. The second phase involved an evaluation of the localization. As mentioned in Section 5.1, we regarded the vehicle position acquired using the state-of-the-art GPS/INS as the ground truth, and calculated the position errors of the data obtained using the smartphone GPS and proposed method. Finally, we compared the localization performance to the LiDAR approaches.

#### 5.2.1. Evaluation of Driving Link Extraction

**Figure 10:**
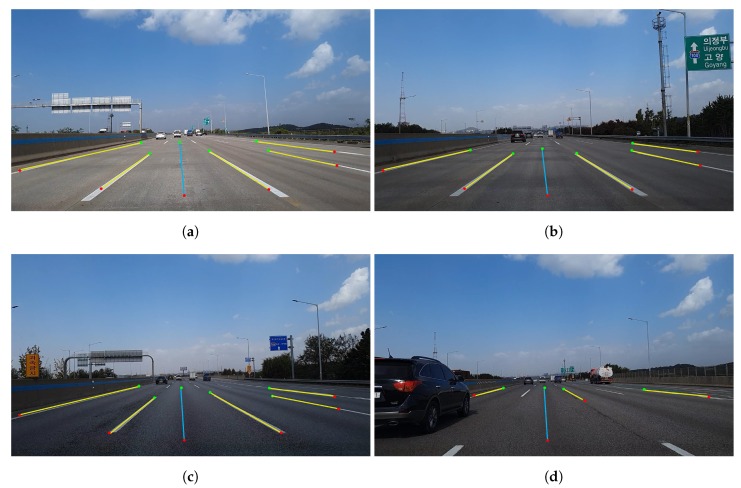
Results of driving link extraction in course 1: (**a**–**c**) correct detection of vehicle lines and extraction of driving link; (**d**) incorrect detection of vehicle lines but update previous driving link.

**Figure 11:**
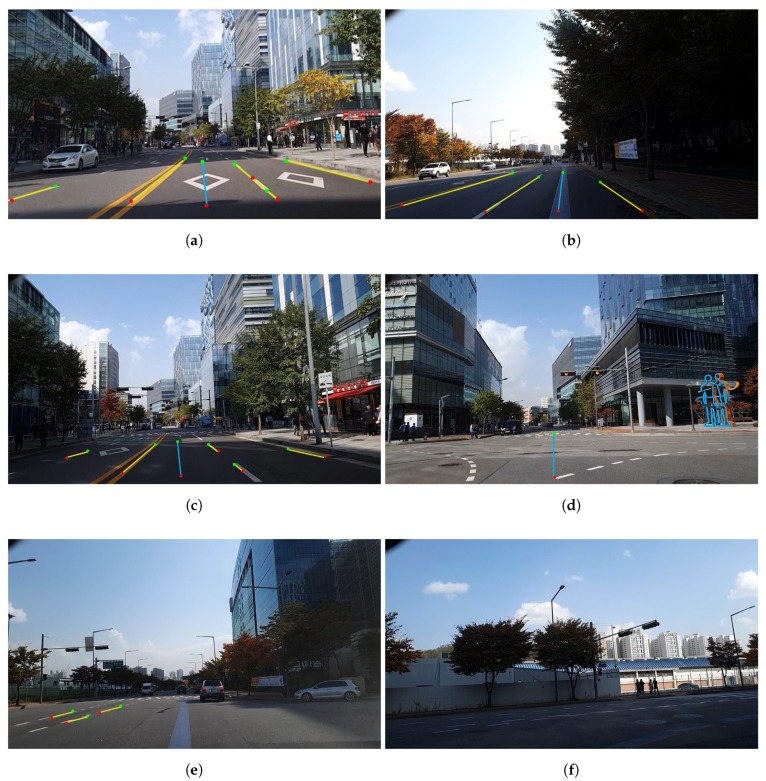
Results of driving link extraction in course 2: (**a**,**b**) correct detection of vehicle lines and extraction of driving link in the changes of illumination; (**c**) correct extraction of driving link by detecting yellow lane marking; (**d**) update previous driving link; (**e**,**f**) extraction failure.

The image acquired from the smartphone camera at the front of the vehicle was 15 fps, and the position information from the GPS was updated at a frequency of 1 Hz. Therefore, after image processing was conducted 15 times, the most duplicated link information was regarded as the current driving link information and was updated at a frequency of 1 Hz. The image set of 6225 frames was selected from course 1, and that of 9450 frames was selected from course 2. The results of the driving link extraction are shown in Figure 10 and Figure 11. Figure 10 shows the results of the driving link extraction in course 1. In the highway area, i.e., course 1, the entire road was separated by a median facility installed with barriers, and all the vehicle lines were marked in white. Therefore, we extracted the driving link information by detecting all vehicle lines in the image frame. We detected all visible vehicle lines in the image, as shown in Figure 10a–c; further, we also extracted the driving link information accurately by calculating the number of vehicle lines from the leftmost line. In course 1, the method failed to detect all the vehicle lines and link information when there was a another vehicle nearby, as shown in Figure 10d. In this case, we updated the previous driving link information as the current information.

Conversely, the urban area, i.e., course 2, had yellow solid lines as the median lane without barriers; thus, the vehicle lines in the opposite direction were detected in certain sections. The median lane detection is essential in image processing to obtain the final driving link information. Because the color of the median lane in urban areas is always yellow, we performed detection of yellow lane marking on the left side of the vehicle lines. When the yellow lane was detected, we labeled the lane as the median lane. The current driving link information depended only on the vehicle lines on the left of the driving direction from the median lane. Figure 11 shows the results of the driving link extraction in course 2. We detected all visible lines in the image despite the changes in illumination, as shown in Figure 11a,b. In Figure 11c, the vehicle lines in the opposite direction were also detected; however, the current driving link information was accurately extracted by detecting the yellow lane marking. It can be observed from Figure 11d that the vehicle lines were not detected at the intersection; however, the driving link information of the previous timestamp was updated with the current information from the constraint. When the vehicle lines for a few timestamps or changes were not detected, extraction failure occurred in certain cases, as shown in Figure 11e,f. However, when the vehicle lines were continuous in the entire course, the correct link information was extracted after normal driving. Table 2 lists the overall results for the driving link extraction.

#### 5.2.2. Evaluation of Localization

**Figure 12:**
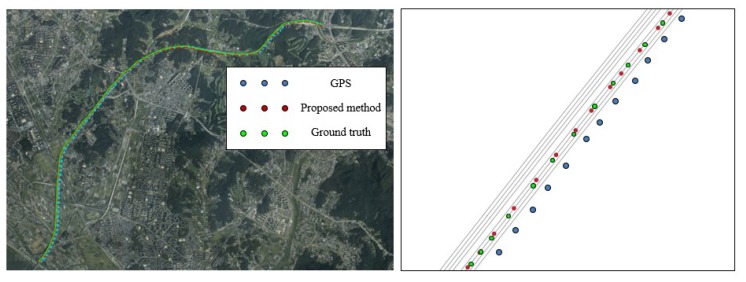
Results of localization in course 1.

**Figure 13:**
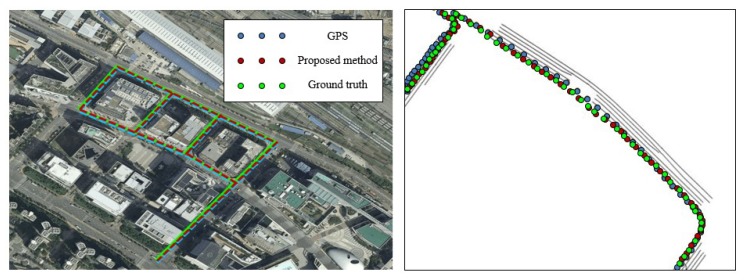
Results of localization in course 2.

After extracting the RMDS from the driving link information, we performed the localization of the map-matching method. We evaluated the localization accuracy by calculating the position errors of the smartphone GPS and the proposed method with respect to the ground truth. The results of the localization are shown in Figure 12 and Figure 13. Each figure contains results of the overall trajectories and the magnified subsections. The mean and standard deviation of the position error distributions are listed in Table 3.

The trajectory of the input GPS followed the shape of the overall road; however, the position accuracy was out of the lane range when compared to the ground truth, as shown in Figure 12. The average error between the position acquired by the state-of-the-art device and that of the GPS was 2.340 m. The position accuracy of the GPS also showed a range within the road; however, it could not provide the lane-level accuracy. Conversely, the trajectory of the proposed method determined the link of the roads on which the vehicle was driven, even if the GPS trajectory was exceeded the lane range. Therefore, the position accuracy was improved via updating of the position on the current driving link. The average square error of the proposed method was 0.475 m, which improved the input GPS location accuracy. Therefore, we performed localization by considering the lane-level accuracy. In the urban areas, as shown in Figure 13, there are buildings and more complex roads than the highway. The average error of the input GPS trajectory was 4.231 m. However, once the driving link was extracted correctly, the position accuracy of the proposed method was improved. The average error of the proposed method was 0.875 m; therefore, it can be observed that we also improved the position accuracy in course 2.

#### 5.2.3. Comparison with LiDAR Approach

**Figure 14:**
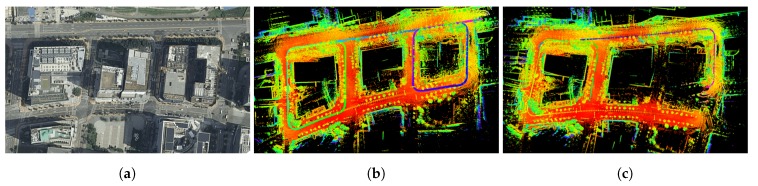
Results of the LiDAR approach in some section of course 2: (**a**) satellite image; (**b**) LiDAR odometry and mapping; (**c**) drift case.

In this section, we compared the localization performance to LiDAR approach. We tested the LeGO-LOAM [11] that detected loop-closures using the ICP algorithm to create a consistent pose graph in some sections of course 2. The total length of the test course was 1.6 km. We utilized our MMS platform which used VLP-16 mounted under the GNSS antenna as shown in Figure 8. The LiDAR data was logged at 10 Hz, and used by method for odometry estimation. The runtime of the odometry was 11.3 ms. To evaluate the localization performance, the position errors was calculated from the LiDAR odometry with respect to the ground truth. First, we initialized the vehicle position to absolute coordinate from sophisticated GPS/INS data. Second, we converted the coordinate of the vehicle position from LiDAR odometry to absolute coordinate at a frequency of 1 Hz based on the initial position. Finally, we evaluated the localization accuracy by calculating the position errors of the estimated position with respect to the ground truth. The error distributions were calculated only in the *x*-*y* plane for comparison with the proposed method. The results of the LiDAR approach are shown in Figure 14, and the position error distributions are listed in Table 4. The LiDAR approach showed the road range localization performance in urban environment. However, this method had a tendency to drift over time in some tests as shown in Figure 14c, and this made it difficult to estimate the vehicle position. In contrast, the position of the vehicle was finally on the HD map, the proposed method did not diverge in the complicated environment.

## 6. Discussion and Conclusions

In this paper, we proposed a map-based localization method using a single camera. In our method, the link information, which indicates the center of each lane, was utilized to achieve lane-level accuracy. In the image processing step, the driving link information was extracted by detecting the vehicle lines in the image frame. The position of the vehicle was estimated via matching of the GPS trajectory and the map information through the ICP-based rigid map-matching method. We performed the experiments using a state-of-the-art GPS/INS device, the data of which was considered as the ground truth. Our experiments demonstrated that the proposed method achieved lane-level position accuracy.

The proposed method fits well to the road condition in South Korea, since we extract the link information from the road markings in compliance with national road regulations. However, if the link information can be extracted from entire road according to the road marking standards of each nation, the proposed map-matching method will be applicable.

Research involving autonomous driving and ADAS requires lane-level accuracy. Several methods have been provided using LiDAR, which is one of the active sensors. However, techniques that utilize LiDAR require high power and have high implementation costs; moreover, they require a large capacity of cloud data. Our method utilized a camera, which is of lower cost when compared to LiDAR; moreover, it achieved accurate determination of lane-level position. Therefore, the proposed method provides an advantage in these fields. In addition, more enhancement in the position accuracy can be anticipated by applying additional sensor fusion to the proposed method.

## Figures and Tables

**Table 1:** Characteristics of experimental courses.

Course	Length (km)	Number of GPS Points	Number of Image Frames
Course 1	10.1	415	6225
Course 2	2.3	630	9450

**Table 2:** Results of link extraction.

Course	Timestamp	Correct Link	Correct Line Rate (%)
Course 1	415	410	98.795
Course 2	630	597	94.761

**Table 3:** Results of localization.

Course	Mean (GPS) (m)	St.Dev. (GPS) (m)	Mean (Prop.) (m)	St.Dev. (Prop.) (m)
Course 1	2.340	1.682	0.475	0.475
Course 2	4.231	1.724	0.875	0.632

**Table 4:** Comparison results.

Method	Mean (m)	St.Dev. (m)
LeGO-LOAM	0.781	0.613
Prop.	0.892	0.781
